# A new model based on multi-axis vision transformer for chondromalacia patella diagnosis in magnetic resonance scans

**DOI:** 10.1007/s13246-026-01707-5

**Published:** 2026-02-04

**Authors:** Semih Demirel, Okan Demirtaş, Sümeyra Kuş Ordu, Ömer Kazcı, Habip Eser Akkaya, Oktay Yıldız

**Affiliations:** 1 Department of Information Systems, Gazi University, Ankara, Turkey; 2 Department of Computer Engineering, Gazi University, Ankara, Turkey; 3 Department of Radiology, Presidential Health Center, Ankara, Turkey; 4 Department of Radiology, Ankara Education and Research Hospital, Ankara, Turkey

**Keywords:** Chondromalacia patella, Vision transformer, Multi-axis vision transformer, Swin transformer, Medical image classification, Deep learning, Convolutional neural networks

## Abstract

A degenerative disease of the patellofemoral joint cartilage, chondromalacia patella (CMP) often results in anterior knee discomfort and functional disability. Determining the best course of therapy and stopping the progression of the disease depend on an accurate and timely diagnosis. In this work, we provide a deep learning architecture based on transformers for the classification of chondromalacia patella using magnetic resonance imaging (MRI) data. We assessed transformer-based designs including Multi-Axis Vision Transformer (MaxViT), Vision Transformer (ViT), and Swin Transformer in addition to convolutional neural network (CNN) based models like Google Network (GoogLeNet), Residual Network 18 (ResNet18), and Mobile Network v2 (MobileNetV2). We evaluated the models’ ability to differentiate between cases of chondromalacia patella and normal cases. With an accuracy of 0.9817, precision of 0.9821, recall of 0.9817, and F1-score of 0.9818, Multi-Axis Vision Transformer outperformed all other models on the testing dataset.

## Introduction

Anterior knee discomfort is frequently the result of chondromalacia patella, a disorder marked by degenerative changes in the patellofemoral joint’s cartilage, one of the most important areas of the knee joint. During or after athletic activities, this condition can cause knee pain, discomfort, and loss of function, especially in young, energetic people [26]. Although cartilage tissue’s structural integrity and ability to reduce friction are essential for joint health, chondromalacia patella’s degenerative nature is highlighted by its low capability for self-healing. Therefore, avoiding increasing cartilage degeneration and identifying suitable treatment options depend on an accurate and timely diagnosis of the illness [31].

Imaging procedures, physical examinations, and clinical examinations are examples of traditional diagnostic approaches. Because it can give high contrast and thorough visualization of cartilage tissue, MRI is specifically regarded as the gold standard for assessing chondromalacia [8]. Despite the fact that MRI enables a thorough examination of the morphological and structural characteristics of the patellofemoral joint’s cartilage, the interpretation of the images frequently results in subjective evaluations due to the requirement for extremely specialized knowledge. This may lead to missed early stage tissue alterations and delays in diagnosis [32]. These drawbacks emphasize the necessity of more impartial and repeatable techniques to aid in the diagnosis procedure.

The development of automated diagnostic systems for medical imaging has advanced significantly in recent years because to ground breaking developments in deep learning and artificial intelligence. Convolutional neural networks and other deep learning models may be trained on big datasets to identify pathological alterations and microscopic tissue distinctions that can be difficult for the human eye to see [22]. These algorithms provide accurate early stage damage identification and dependable disease stage categorization, especially in the evaluation of patellar cartilage [37]. In this regard, techniques based on deep learning have a lot of promise for expediting clinical diagnostic procedures and facilitating impartial assessments.

Created algorithms using deep learning are currently being used to diagnose a variety of illnesses after being combined with information gleaned from MR images. These techniques’ capacity to accurately identify cartilage degradation at various clinical phases is demonstrated by their training on data from sizable patient populations. Furthermore, they have a major advantage in guiding patients toward early intervention and therapy because to their high sensitivity and specificity in detecting microscopic tissue alterations at an early stage [15, 36]. Therefore, the possibility for better disease progression assessment and treatment planning is increased by the development of automated and objective diagnostic tools in both clinical and research settings.

The study by [5] used a variety of classification techniques and vibroarthrographic (VAG) signals to distinguish between normal instances and knee joint problems. After processing the signals, features were taken out. Several machine learning techniques were used to categorize knee joint diseases following extraction. A least squares support vector machine (LSSVM) with the apriori method produced the best accuracy rate of 0.9431.

Vibroarthrography signals were used in [19] to classify osteoarthritis. They evaluated 84 patients, 40 of whom were in the research group and 40 of whom were in the control group. Machine learning methods were employed to categorize osteoarthritis. A multilayer perceptron produced the greatest results, with an accuracy rate of 0.893.

X-ray, computer tomography (CT), and MRI were among the imaging modalities used to classify anomalies in the knee in the study by [30]. Using an ensemble approach, the suggested network produced three outcomes from pictures acquired using various modalities. After the images’ features were taken out, they were put into an attention-based convolutional neural network. They obtained an F1-score of 0.85 and an accuracy rate of 0.84.

[27] examined knee vibroarthrographic signals in order to determine possible parameters for the early identification of patellar chondromalacia. Analysis was done on the signals’ frequency properties, such as energy and the total spectral power in each 500 Hz frequency range. They discovered that, especially in the high-frequency bands, the energy and spectral power of VAG signals in people with chondromalacia patella were noticeably greater than those of normal VAG signals.

The work by [33] created a novel technique that uses texture analysis of magnetic resonance images and cartigrams. The study looked at 101 people, 65 of whom had chondromalacia patella and 36 of whom were controls, to assess patellar cartilage and its relationship with femorotibial cartilage. Six prediction models were used to extract and assess 43 femorotibial cartilage textures and 27 patellar cartilage cartigram characteristics. Using cartigrams in the deep layer produced the greatest results for patellar cartilage, with an average AUC of 0.75 using the linear support vector machine model and three features.

A computer-assisted diagnostic technique was developed in the study by [40] to identify osteoarthritis in the knee using arthrogram signals. They suggested a deep learning model that combines an aggregated multiscale dilated convolution network with a laplace distribution-based approach in order to do this. The accuracy rate they obtained with the suggested network was 0.936.

The work by [39] sought to investigate and assess a new technique for using radiomic characteristics from patellar sagittal T2-weighted (T2W) images to diagnose patellar chondromalacia. Sagittal T2W scans of the patella from 40 patients with patellar chondromalacia and 40 healthy individuals were included in the experimental data. The categorization model was trained using a machine learning technique and subsequently assessed. The accuracy of the testing set was 0.7.

Our work is distinct from previous research since it uses MRI scans to categorize chondromalacia patella and normal instances. Prior research in the literature has mostly concentrated on utilizing vibroarthrographic signals to diagnose osteoarthritis or identify anomalies in the knee. Nevertheless, there aren’t many research that focus on chondromalacia patella identification particularly. Our work offers a unique contribution to the literature in this respect.

Global structural features including cartilage thickness, morphological integrity, or tissue degeneration are often the focus of MRI-based cartilage evaluation techniques in the literature. Nevertheless, no deep learning-based study that explicitly targets the identification of chondromalacia patella from MRI scans has been found, according to a thorough analysis of the literature. Research in this field has also been limited by the absence of a publically accessible MRI dataset for CMP categorization.

As a consequence, MRI images were carefully gathered in this study in compliance with conventional clinical MRI protocols and annotated in line with expert opinions, creating a new dataset not seen in the existing literature. Due to the small quantity of data available, the current study was intended as a starting step focused on binary classification, even though examining CMP across four different grades would be clinically more thorough and useful.

Using information from MRI scans, a deep learning-based model was created for this work, and its ability to diagnose chondromalacia patella was carefully assessed. The study’s main goals are to provide a new paradigm for the diagnosis of chondromalacia patella and show how deep learning algorithms can be used to evaluate the cartilage tissue in the knee joint.

This was accomplished by using CNN-based architectures, such as GoogLeNet [41], ResNet18 [17], and MobileNetV2 [35], as well as transformer-based models, such as MaxViT [42], ViT [11], and the Swin Transformer [24], for the binary classification of chondromalacia patella and normal cases. The findings show that deep learning algorithms are capable of accurately and successfully identifying chondromalacia patella.

The following are the main contributions of our study:

- A novel dataset of MRI images was produced for the identification of chondromalacia patella.
- The transformer and CNN models were thoroughly compared on the training, validation, and testing datasets.
- A thorough performance comparison and statistical analysis were also carried out.
- Our goal is to detect chondromalacia patella in MRI images, as opposed to the studies in the literature that focus on knee anomalies.

The following is the rest of the paper: The dataset and transformer-based deep learning models are described in depth in Sect. 2. Section 3 presents the models’ experimental results. The experimental results are thoroughly evaluated and interpreted in Sect. 4. Section 5 provides a detailed analysis of the research findings.

## Methods

### Magnetic resonance imaging protocol

A Siemens 1.5 Tesla MRI scanner was used to obtain the knee MRI images used in this investigation. The following settings were made for the imaging parameters: T2-weighted and proton density (PD) sequences were among the imaging sequences. The field of view (FOV) was adjusted to 16 cm, the matrix size to 512 $\times$ 512, and the slice thickness to 3 mm. For the T2 sequence, the repetition duration (TR) fluctuated between 2000 and 4000 ms, while the time-to-echo (TE) values ranged from 40 to 80 ms. The axial, coronal, and sagittal planes of the images were examined. Every image utilized in the study was obtained in the axial plane; no images from other imaging planes were mixed together. The axial view offers the best visibility for the evaluation of chondromalacia patella, which is the main reason for this decision.

### Dataset

The relevant medical center’s standard clinical procedures provided the joint MRI images used in this investigation. The study was carried out with the Gazi University Ethics Committee’s consent; on December 13, 2024, an official ethics consent document (Approval No: 1117365) was given.

The details of the dataset are presented in the Table 1.

**Table 1:** Dataset details

		Training	Validation	Testing	Total
Fold1	Grade0	306	34	85	425
	Grade1-2-3-4	754	84	210	1048
Fold2	Grade0	306	34	85	425
	Grade1-2-3-4	754	84	210	1048
Fold3	Grade0	306	34	85	425
	Grade1-2-3-4	754	84	210	1048
Fold4	Grade0	306	34	85	425
	Grade1-2-3-4	754	84	210	1048
Fold5	Grade0	306	34	85	425
	Grade1-2-3-4	754	84	210	1048

The 5-fold cross validation method was used to divide the dataset into training and testing sets. 10% of the training data was then divided for validation. There are 1048 image files from the Grade 1–4 classes and 425 images from the Grade 0 class in each fold, for a total of 1473 images in the dataset.

In every experiment, a strict data partitioning procedure was used to avoid any data leaking during the data splitting phase. A 5-fold cross validation strategy was used to arrange the training data. In each fold, the data were divided into training and testing sets, with an extra 10% of the training part put aside as a validation set. Testing and validation samples were kept out of the training process at all times thanks to this strategy. In order to avoid enhanced samples from spreading to other splits and creating almost identical instances, data augmentation techniques were only applied to the training set. The data splitting was naturally carried out at the patient level since the dataset does not contain many images from the same patient; as a result, no image from a particular patient appears in more than one set. Additionally, only the training data was used for hyperparameter tuning; neither the validation nor the testing sets were utilized for model selection or hyperparameter optimization. The testing set was kept completely separated under this technique, guaranteeing that the stated performance metrics were acquired apart from the training procedure.

Low-quality or unreadable images were removed prior to analysis. Furthermore, to minimize error margins and ensure diagnostic accuracy, all images were first reviewed by a medical expert and then verified by a second expert. All knee MRI examinations were independently reviewed for the presence and grading of chondromalacia patella by two radiologist-researchers, each with more than 10 years of subspecialty experience in musculoskeletal imaging. Initial readings were performed separately and blinded to all clinical data. In cases of discrepant interpretations, the images were re-evaluated in a dedicated consensus session to establish a final agreed result. The inter-researchers reliability of the assessments was excellent, with a Cohen’s kappa coefficient exceeding 0.91, indicating a very high level of consistency between the two readers.

**Fig. 1:**
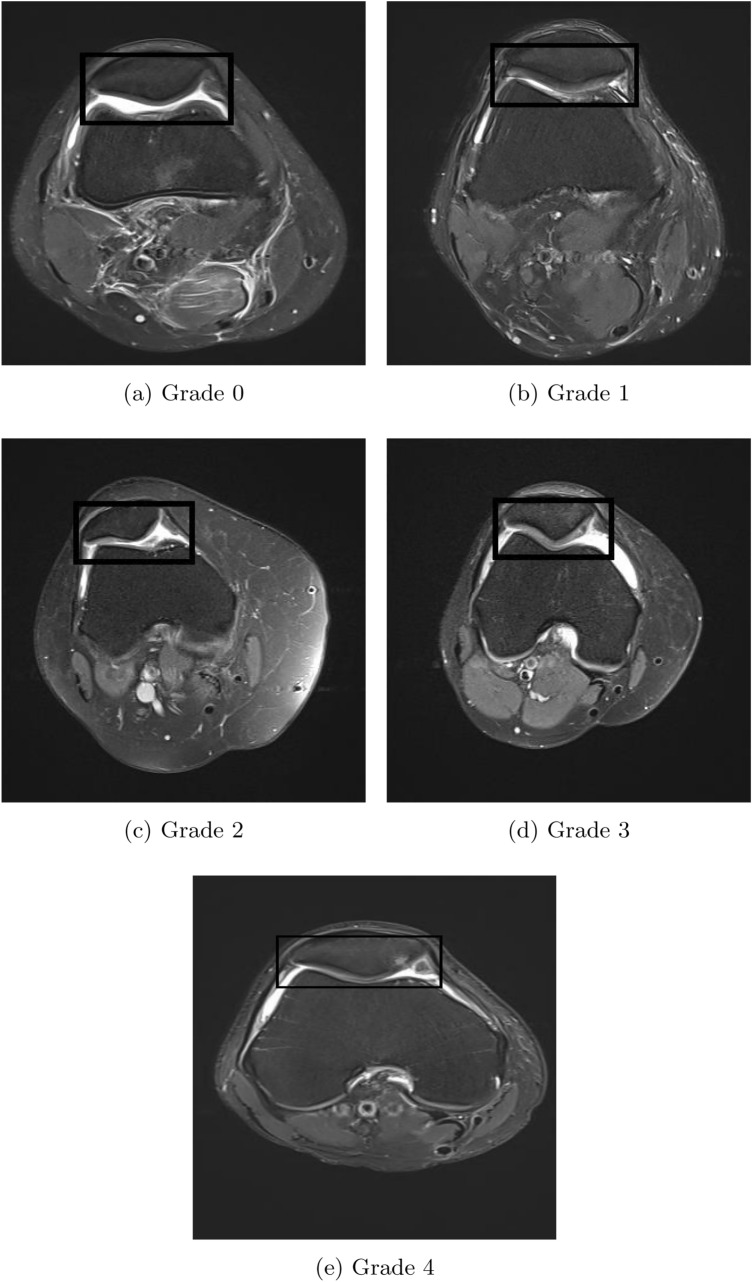
The dataset’s examples of images are displayed. A medical professional annotates every image with the appropriate grade. Normal instances are represented by grade 0, while CMP cases are represented by grades 1-4

The dataset’s sample images are displayed in Fig. 1.

### GoogLeNet

Increasing the network’s depth and width for greater accuracy while maintaining computational efficiency was the main driving force behind GoogLeNet. The core component of GoogLeNet is the Inception module [4]. The Inception module performs a max pooling operation in addition to multiple convolution processes with filter sizes of 1 $\times$ 1, 3 $\times$ 3, and 5 $\times$ 5 in at the same time [16]. After that, the outputs from each of these parallel channels are concatenated depthwise. Since they have the significant computational cost, $1 \times 1$ convolutions are employed before layers. The input feature map’s channel size is decreased by the $1 \times 1$ convolution [7].

The input feature map is represented by Eq. 1.

1 $$\begin{aligned} X \in \mathbb {R}^{H \times W \times C} \end{aligned}$$

where, *X* is the input feature map.

The four parallel branches that make up the Inception module are described by Eq. 2.

2 $$\begin{aligned} \begin{aligned} Y_1&= f_{1\times 1}(X) \\ Y_2&= f_{3\times 3}\big (f_{1\times 1}(X)\big ) \\ Y_3&= f_{5\times 5}\big (f_{1\times 1}(X)\big ) \\ Y_4&= f_{1\times 1}\big (p_{3\times 3}(X)\big ) \end{aligned} \end{aligned}$$

where, a convolution process with a $k \times k$ kernel and a nonlinear activation function is shown by $f_{k\times k}()$, $p_{3\times 3}()$ denotes a max-pooling operation of $3 \times 3$.

The output of the Inception module is obtained by concatenating the outputs of every branch along the channel dimension, as shown in Eq. 3.

3 $$\begin{aligned} Y_{\text {Inception}} = \operatorname {Concat} \left( Y_1, Y_2, Y_3, Y_4 \right) \end{aligned}$$

where $\operatorname {Concat}()$ denotes the feature maps’ channel-wise concatenation.

**Fig. 2:**
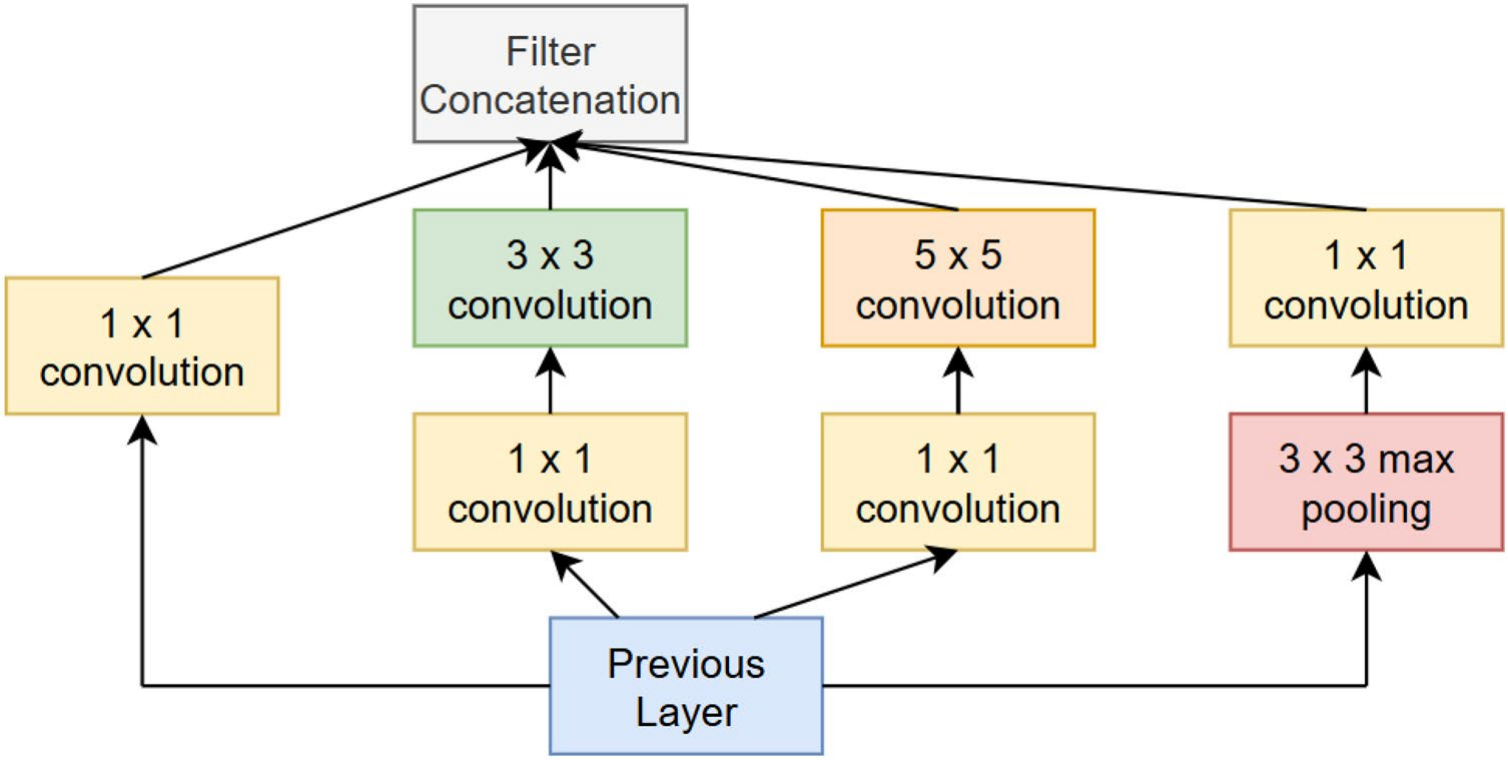
Inception module that uses 1 x 1 convolution [41]

The 1 $\times$ 1 convolution with Inception module is shown in Fig. 2.

### Swin transformer

A hierarchical vision transformer architecture-based on windowed self-attention is shown by the Swin Transformer [25]. A shifted-window multi head self-attention mechanism facilitates cross window information flow, while window multi head self-attention computes self-attention inside non-overlapping local windows [23]. Layer normalization and residual connections are wrapped around a multilayer perceptron, which comes after alternating window multi head self-attention and shifted window multi head self-attention layers in each Swin block [38]. The Swin Transformer can effectively scale to high resolution vision tasks thanks to its hierarchical structure and linear computing complexity.

**Fig. 3:**
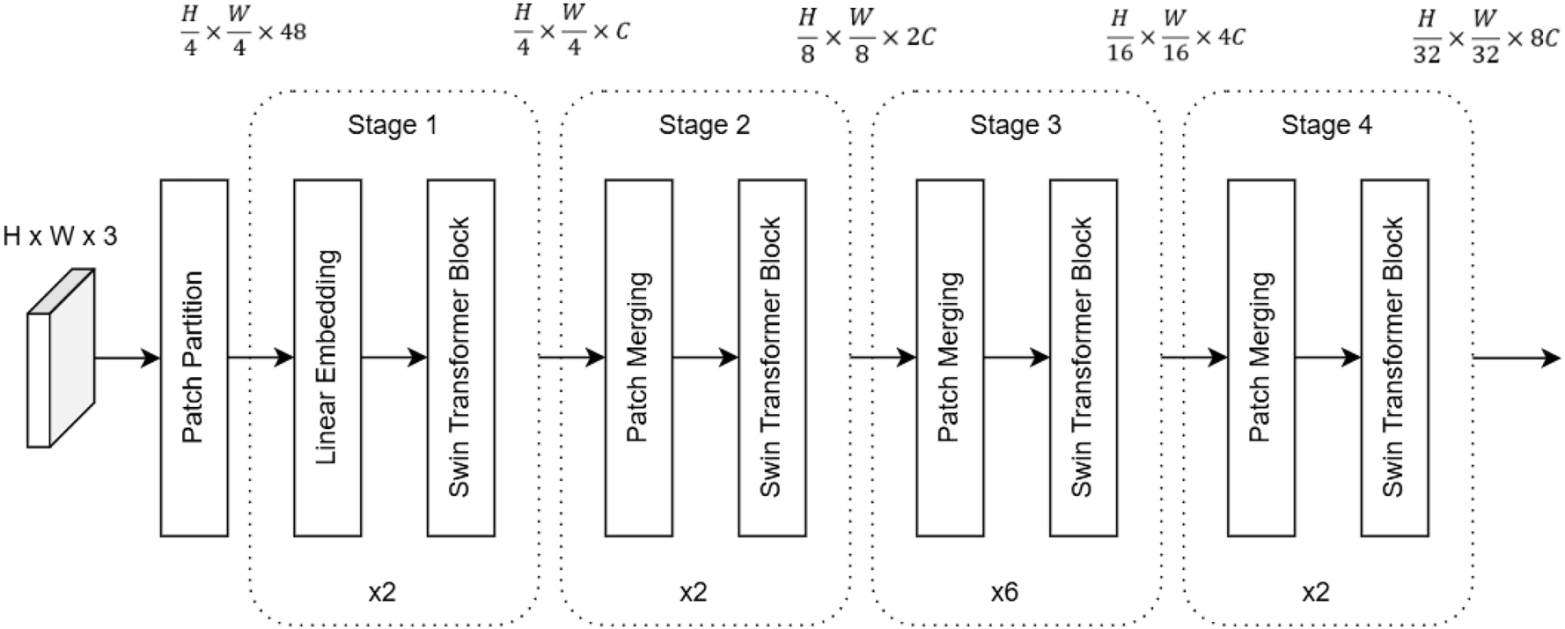
Architecture of Swin Transformer [24]

The Swin Transformer’s architecture is displayed in Fig. 3.

### ResNet18

ResNet18 is a lightweight residual convolutional neural network with 18 layers structured into four basic block phases. By using identity shortcut connections, each basic block helps the model acquire residual mappings, which reduces vanishing gradients and enhances training stability [47]. ResNet18 offers a strong foundation for image classification and other downstream applications thanks to its low parameter count, hierarchical feature extraction, and excellent residual architecture [3].

Given an input feature map, $X \in \mathbb {R}^{H \times W \times C}$, Eq. 4 defines the output of a residual block.

4 $$\begin{aligned} y = \mathcal {F}(x) + X \end{aligned}$$

**Fig. 4:**
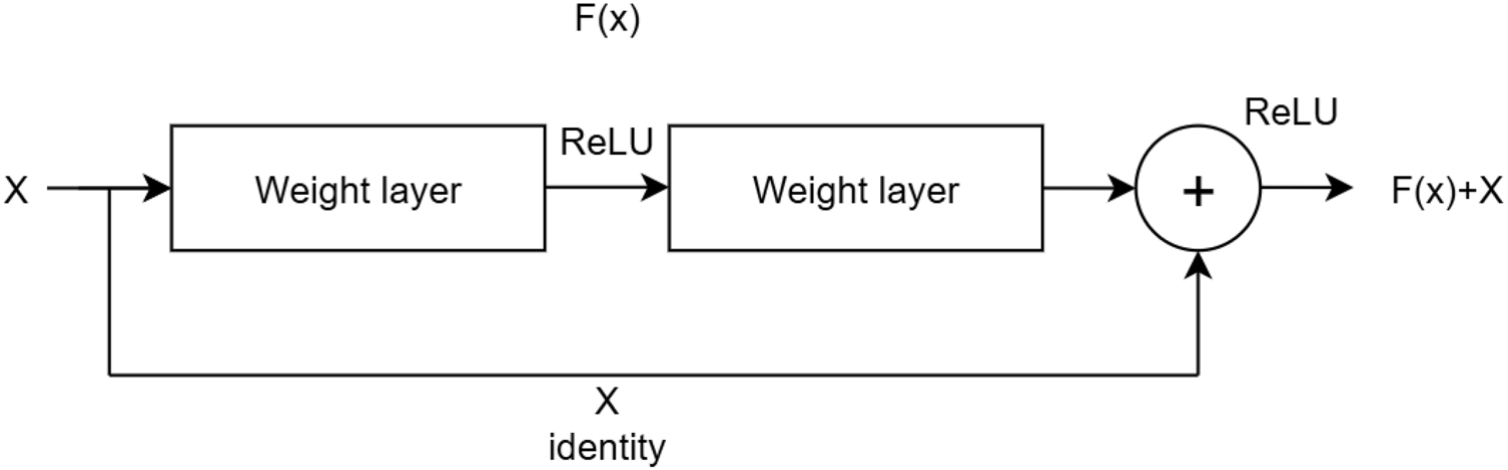
Residual block of ResNet18 [17]

Figure 4 provides an illustration of a residual block.

### MobileNetV2

In order to achieve great efficiency in mobile and embedded vision workloads, MobileNetV2 incorporates the inverted residual and linear bottleneck architecture [18]. Each block uses a 1x1 convolution to extend the input channels, a depthwise 3x3 convolution, and a linear 1x1 convolution to project the features back to a low-dimensional space [29]. When the input and output resolutions occurs residual connections are used. This approach minimizes computing expense while maintaining representational power.

Table 2 provides the MobileNetV2’s tabular form.

**Table 2:** MobileNetV2 architecture with an input size of $224 \times 224 \times 3$ [35]

Stage	Operator	*t*	*c*	*n*	*s*
1	Convolution $3 \times 3$	–	32	1	2
2	Bottleneck	1	16	1	1
3	Bottleneck	6	24	2	2
4	Bottleneck	6	32	3	2
5	Bottleneck	6	64	4	2
6	Bottleneck	6	96	3	1
7	Bottleneck	6	160	3	2
8	Bottleneck	6	320	1	1
9	Convolution $1 \times 1$	–	1280	1	1
10	GAP + FC	–	Classes	1	–

In Table 2, *t* is the expansion factor that regulates the channel-wise expansion within each inverted residual block through the initial $1 \times 1$ convolution, *c* is the number of output channels generated by the projection layer, *n* is the number of repeated blocks in a stage, and *s* is the first block’s stride, which establishes the feature maps’ spatial downsampling [13]. The final classification head is represented by the fully connected (FC) and global average pooling (GAP) layers, which combine spatial feature maps into a compact feature vector.

### Vision transformer

**Fig. 5:**
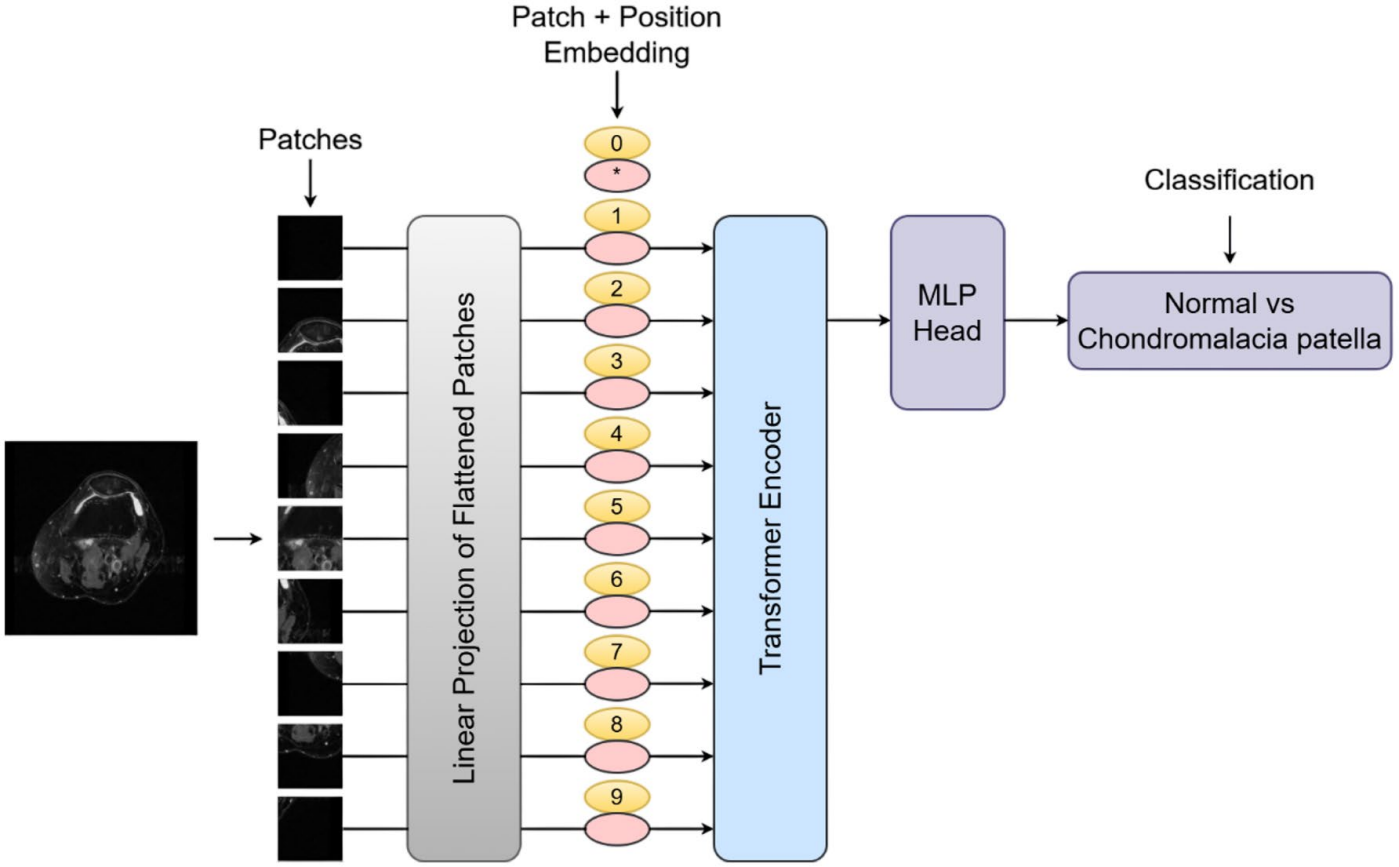
Architecture of Vision Transformer [11]

Images are divided into fixed-size patches by ViT, which interprets them as a sequence of tokens [12]. An input image $\textbf{x} \in \mathbb {R}^{H \times W \times C}$ is used to construct non-overlapping patches of size $P \times P$, where *H*, *W* represent the image height and width, *C* represents the number of channels, and *P* represents the patch size. Figure 5 displays the Vision Transformer’s construction.

A linear projection is used to embed each patch into a D-dimensional space [1]. The linear projection formula is $Z=x_pE$, where *E* is a learnable projection matrix and *Z* is the resultant patch embeddings.

A positional encoding $Z_0=Z+P$, where *P* is a learnable positional encoding, is used to create spatial information while preserving spatial structure [46].

ViT employs a transformer encoder that combines multi-head self-attention with feedforward networks. Using learnable weight matrices in multi-head self attention, each embedding *Z* is transformed into queries (Q), keys (K), and values (V) using equation 5 [34].

5 $$\begin{aligned} Q = ZW_Q, K=ZW_K, V=ZW_V \end{aligned}$$

Using Eq. 6, the scaled dot-product attention is calculated.

6 $$\begin{aligned} Attention(Q, K, V) = softmax(\frac{QK^T}{\sqrt{d_k}})V \end{aligned}$$

Equation 7 defines multi-head attention.

7 $$\begin{aligned} MHSA(Z) = Concat(head_1, ..., head_h)W_o \end{aligned}$$

where the output projection is $W_o \in \mathbb {R}^{hd_k \times D}$. After the transformer block, the class token is retrieved and then classified using a multilayer perceptron.

### Multi-axis vision transformer

For effective feature extraction, MaxViT uses mobile inverted bottleneck convolution (MBConv) blocks [28]. Equation 8 expresses an MBConv block without downsampling given an input feature map $\textbf{x} \in \mathbb {R}^{H \times W \times C}$.

8 $$\begin{aligned} \textbf{x} = \textbf{x} + \text {Proj}(\text {SE}(\text {DWConv}(\text {Conv}(\text {Norm}(\textbf{x}))))), \end{aligned}$$

Batch normalization is denoted by *Norm*, channel expansion by *Conv*, depthwise $3 \times 3$ convolution by *DWConv*, squeeze-and-excitation layer by *SE*, and channel reduction by *Proj*, which is pointwise $1 \times 1$ convolution.

To improve spatial awareness, MaxViT uses relative attention [14]. Equation 9 defines the self-attention mechanism given query $\textbf{Q}$, key $\textbf{K}$, value $\textbf{V}$, and a learned positional bias $\textbf{B}$.

9 $$\begin{aligned} \text {RelAttention}(\textbf{Q}, \textbf{K}, \textbf{V}) = \text {softmax}\left( \frac{\textbf{Q} \textbf{K}^T}{\sqrt{d}} + \textbf{B} \right) \textbf{V}, \end{aligned}$$

where *d* represents the hidden dimension.

Block Attention functions locally inside $P \times P$ image blocks that do not overlap [42]. Grid attention uses sparse global attention to capture long-range interdependence.

Equation 10 is defined in [42] to divide an input feature $\textbf{x} \in \mathbb {R}^{H \times W \times C}$ into blocks in block attention.

10 $$\begin{aligned} & \text {Block}: (H, W, C) \rightarrow \left( \frac{H}{P} \times P, \frac{W}{P} \times P, C \right) \nonumber \\ & \quad \rightarrow \left( \frac{HW}{P^2}, P^2, C \right) \end{aligned}$$

Each block’s self-attention is applied using Eq. 11.

11 $$\begin{aligned} \textbf{x} = \textbf{x} + \text {Unblock}(\text {RelAttention}(\text {Block}(\text {LN}(\textbf{x})))) \end{aligned}$$

where the inverse operations of the block functions are denoted by *unblock*. The grid partitioning for global feature extraction is described by Eq. 12 [42].

12 $$\begin{aligned} \text {Grid} : (H, W, C) \rightarrow (G \times \frac{H}{G}, G \times \frac{W}{G}, C) \rightarrow (G^2, \frac{HW}{G^2}, C). \end{aligned}$$

The self-attention inside each grid sector is applied using Eq. 13.

13 $$\begin{aligned} \textbf{x} = \textbf{x} + \text {Ungrid}(\text {RelAttention}(\text {Grid}(\text {LN}(\textbf{x})))) \end{aligned}$$

where the inverse operations of the grid functions are denoted by *ungrid*.

**Fig. 6:**
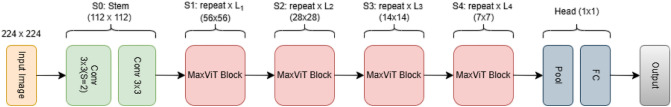
Architecture of MaxViT model [42]

The MaxViT model’s design is displayed in Fig. 6.

**Fig. 7:**
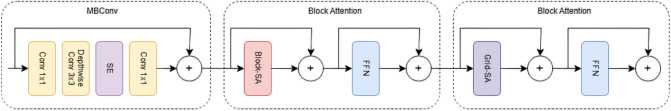
Architecture of MaxViT block [42]

The MaxViT block’s design is depicted in Fig. 7.

## Results

For this study, training and testing were conducted using the Windows 11 operating system. The specifics of computer hardware are as follows: Technical specifications include an Intel Core i5-11320 H central processing unit (CPU) running at 3.2 GHz, a 4 GB (gigabyte) Nvidia Geforce 3050Ti graphic processing unit (GPU), and 8 GB of random access memory (RAM).

Equations 14, 15, 16, and 17 were used to evaluate the model performance. In addition, thorough statistical analyses were carried out and the McNemar’s p-value and bootstrapped confidence intervals (CIs) were calculated.

14 $$\begin{aligned} accuracy = \frac{TP + TN}{TP + FP + TN + FN} \end{aligned}$$

Accuracy gives an overall performance evaluation by calculating the percentage of properly identified samples among all samples.

15 $$\begin{aligned} precision = \frac{TP}{TP + FP} \end{aligned}$$

Precision measures the accuracy of positive predictions by dividing the number of accurately predicted positive samples by the total number of expected positives.

16 $$\begin{aligned} recall = \frac{TP}{TP + FN} \end{aligned}$$

Recall determines how effectively the model catches positive instances by calculating the percentage of real positive samples that were accurately recognized.

17 $$\begin{aligned} f1 = 2 \times \frac{precision \times recall}{precision + recall} \end{aligned}$$

The F1-score is a statistic that balances precision and recall by taking the harmonic mean of the two. It ensures that neither false positives nor false negatives dominate the evaluation, which is particularly helpful when there is an imbalance between positive and negative classifications.

95% confidence intervals were produced for whole measures in order to evaluate the dependability of the estimated performance metrics. An estimated range that is anticipated to include the real value of a metric with 95% certainty is provided by a 95% confidence interval. While narrow intervals show more consistent and dependable model performance, wide intervals show greater variability and less assurance in the measure. The bootstrap resampling method, which repeatedly samples the testing predictions with replacement to mimic the sampling distribution of each measure, was used in this work to establish confidence intervals.

To ascertain if the performance difference between two classification models is statistically significant, the McNemar test was used in addition to confidence interval calculation. The McNemar test is especially intended to compare two models assessed on the same testing set. It concentrates on discordant pairings, or instances when one model incorrectly classifies but the other properly classifies. The test yields a p-value, which indicates that the performance difference is unlikely to have happened by coincidence if it is less than the widely accepted cutoff of 0.05.

The training process was carried out using the PyTorch framework. The Adam [20] optimizer and an initial learning rate of 0.0001 were used to train each model for a total of 200 epochs. The batch size was set to 4 in order to guarantee that the model fit within the available memory because the machine utilized in this investigation had an RTX 3050 Ti GPU with 4 GB of video random access memory (VRAM). The input images were resized to 224 $\times$ 224 pixels before being fed into the models. The images were also normalized using the ImageNet [10] dataset’s mean and standard deviation values.

The dataset shows a significant class imbalance between the normal and chondromalacia patella classes. During the training phase, several strategies were integrated to lessen the possible influence of this imbalance on model performance. First, focal loss [21] was used, which is well-known for its ability to address class imbalance. Focal loss, which was first introduced in the RetinaNet [21] study, improves minority class representation during training while decreasing majority class dominance.

To improve the model’s capacity for generalization and raise the effective sample diversity, data augmentation approaches were also used. All training samples were treated to the following changes with equal chance using the PyTorch DataLoader: random rotation (±20$^{\circ }$), random horizontal flip, random vertical flip, and color jitter (brightness=0.2, contrast=0.2, saturation=0.2). By increasing the training set’s diversity regardless of class distribution, these augmentations lessened the possibility of overfitting.

Dynamic training control techniques based on validation performance were used to preserve training stability and avoid the model overfitting to the majority class. The learning rate was lowered by a factor of 0.5 when validation accuracy did not increase for five consecutive epochs. Early stopping was initiated if no progress was seen after fifteen epochs. By avoiding needless training, this approach lessens the possibility of overly adapting to the majority class. The checkpoint with the best validation accuracy was chosen as the final model, and model weights were recorded at the end of each epoch.

### Experimental findings

Table 3 displays the experimental results of the categorization of normal and chondromalacia patella on the training dataset.

**Table 3:** Training results

Fold	Model	Accuracy	Precision	Recall	F1-Score
Fold1	MaxViT	0.9972	0.9972	0.9972	0.9972
	ResNet18	0.9934	0.9935	0.9934	0.9934
	Swin Transformer	0.9943	0.9943	0.9943	0.9943
	ViT	0.9953	0.9953	0.9953	0.9953
	GoogLeNet	1.0000	1.0000	1.0000	1.0000
	MobileNetV2	0.9981	0.9981	0.9981	0.9981
Fold2	MaxViT	0.9981	0.9981	0.9981	0.9981
	ResNet18	0.9981	0.9981	0.9981	0.9981
	Swin Transformer	1.0000	1.0000	1.0000	1.0000
	ViT	0.9679	0.9689	0.9679	0.9682
	GoogLeNet	1.0000	1.0000	1.0000	1.0000
	MobileNetV2	1.0000	1.0000	1.0000	1.0000
Fold3	MaxViT	0.9981	0.9981	0.9981	0.9981
	ResNet18	0.9991	0.9991	0.9991	0.9991
	Swin Transformer	0.9887	0.9889	0.9887	0.9887
	ViT	0.9915	0.9916	0.9915	0.9915
	GoogLeNet	1.0000	1.0000	1.0000	1.0000
	MobileNetV2	0.9991	0.9991	0.9991	0.9991
Fold4	MaxViT	0.9981	0.9981	0.9981	0.9981
	ResNet18	0.9943	0.9944	0.9943	0.9943
	Swin Transformer	1.0000	1.0000	1.0000	1.0000
	ViT	0.9604	0.9604	0.9604	0.9604
	GoogLeNet	1.0000	1.0000	1.0000	1.0000
	MobileNetV2	0.9991	0.9991	0.9991	0.9991
Fold5	MaxViT	1.0000	1.0000	1.0000	1.0000
	ResNet18	0.9915	0.9917	0.9915	0.9915
	Swin Transformer	1.0000	1.0000	1.0000	1.0000
	ViT	0.9972	0.9972	0.9972	0.9972
	GoogLeNet	1.0000	1.0000	1.0000	1.0000
	MobileNetV2	0.9981	0.9981	0.9981	0.9981
Average	MaxViT	0.9983	0.9983	0.9983	0.9983
	ResNet18	0.9953	0.9954	0.9953	0.9953
	Swin Transformer	0.9966	0.9966	0.9966	0.9966
	ViT	0.9825	0.9827	0.9825	0.9825
	GoogLeNet	1.0000	1.0000	1.0000	1.0000
	MobileNetV2	0.9989	0.9989	0.9989	0.9989

The 5-fold cross validation’s average training results show that every model performed very well across evaluation metrics. GoogLeNet has an exceptionally high fitting capacity among the architectures assessed, with perfect average scores of 1.0000 across all measures. Additionally, MobileNetV2, MaxViT, Swin Transformer, and ResNet18 demonstrate steady convergence and efficient feature learning with consistently high accuracy and low variability between folds. The ViT, on the other hand, performs the worst on average, especially in folds 2 and 4.

Table 4 presents the experimental results of the validation dataset.

**Table 4:** Validation Results

Fold	Model	Accuracy	Precision	Recall	F1-Score
Fold1	MaxViT	1.0000	1.0000	1.0000	1.0000
	ResNet18	1.0000	1.0000	1.0000	1.0000
	Swin Transformer	0.9661	0.9661	0.9661	0.9661
	ViT	0.9831	0.9831	0.9831	0.9831
	GoogLeNet	0.9831	0.9831	0.9831	0.9831
	MobileNetV2	1.0000	1.0000	1.0000	1.0000
Fold2	MaxViT	0.9576	0.9601	0.9576	0.9581
	ResNet18	0.9746	0.9766	0.9746	0.9749
	Swin Transformer	0.9661	0.9697	0.9661	0.9666
	ViT	0.9322	0.9339	0.9322	0.9328
	GoogLeNet	0.9576	0.9601	0.9576	0.9581
	MobileNetV2	0.9661	0.9697	0.9661	0.9666
Fold3	MaxViT	0.9746	0.9745	0.9746	0.9745
	ResNet18	0.9831	0.9831	0.9831	0.9831
	Swin Transformer	0.9237	0.9230	0.9237	0.9226
	ViT	0.9576	0.9581	0.9576	0.9578
	GoogLeNet	0.9746	0.9766	0.9746	0.9749
	MobileNetV2	0.9492	0.9506	0.9492	0.9496
Fold4	MaxViT	0.9492	0.9492	0.9492	0.9492
	ResNet18	0.9746	0.9750	0.9746	0.9747
	Swin Transformer	0.9661	0.9661	0.9661	0.9661
	ViT	0.9237	0.9230	0.9237	0.9226
	GoogLeNet	0.9831	0.9831	0.9831	0.9831
	MobileNetV2	0.9915	0.9918	0.9915	0.9916
Fold5	MaxViT	0.9831	0.9831	0.9831	0.9831
	ResNet18	0.9746	0.9750	0.9746	0.9747
	Swin Transformer	0.9915	0.9918	0.9915	0.9916
	ViT	0.9576	0.9574	0.9576	0.9574
	GoogLeNet	0.9746	0.9750	0.9746	0.9747
	MobileNetV2	0.9492	0.9489	0.9492	0.9487
Average	MaxViT	0.9729	0.9734	0.9729	0.9730
	ResNet18	0.9814	0.9819	0.9814	0.9815
	Swin Transformer	0.9627	0.9633	0.9627	0.9626
	ViT	0.9508	0.9511	0.9508	0.9507
	GoogLeNet	0.9746	0.9756	0.9746	0.9748
	MobileNetV2	0.9712	0.9722	0.9712	0.9712

All models show good generalization performance, but with more variability than during the training phase, according to the validation findings compiled in Table 4. With an accuracy of 0.9814, ResNet18 outperforms the other analyzed architectures on average. GoogLeNet comes in second with an accuracy of 0.9746, MaxViT with an accuracy of 0.9729, and MobileNetV2 with an accuracy of 0.9712. These models demonstrate robust classification performance throughout validation folds by consistently producing excellent accuracy, recall, and F1-score values. With an average accuracy of 0.9627, the Swin Transformer performs moderately, while the ViT has the lowest average accuracy of 0.9508.

The experimental findings of evaluating the testing dataset for chondromalacia patella and normal are given in Table 5.

**Table 5:** Testing results with 95% confidence intervals

Fold	Model	Accuracy/95% CI	Precision/95% CI	Recall/95% CI	F1-Score/95% CI
Fold1	MaxViT	0.9797/(0.9627, 0.9932)	0.9804/(0.9650, 0.9934)	0.9797/(0.9627, 0.9932)	0.9798/(0.9631, 0.9932)
	ResNet18	0.9763/(0.9559, 0.9932)	0.9768/(0.9596, 0.9932)	0.9763/(0.9559, 0.9932)	0.9764/(0.9596, 0.9932)
	Swin Transformer	0.9763/(0.9593, 0.9932)	0.9763/(0.9591, 0.9902)	0.9763/(0.9559, 0.9932)	0.9761/(0.9557, 0.9932)
	ViT	0.9695/(0.9492, 0.9864)	0.9694/(0.9490, 0.9867)	0.9695/(0.9492, 0.9864)	0.9694/(0.9487, 0.9865)
	GoogLeNet	0.9831/(0.9661, 0.9966)	0.9831/(0.9671, 0.9966)	0.9831/(0.9661, 0.9966)	0.9831/(0.9665, 0.9966)
	MobileNetV2	0.9695/(0.9492, 0.9864)	0.9700/(0.9502, 0.9870)	0.9695/(0.9492, 0.9864)	0.9696/(0.9495, 0.9865)
Fold2	MaxViT	0.9831/(0.9661, 0.9966)	0.9831/(0.9664, 0.9966)	0.9831/(0.9661, 0.9966)	0.9831/(0.9663, 0.9966)
	ResNet18	0.9831/(0.9661, 0.9966)	0.9840/(0.9721, 0.9966)	0.9831/(0.9661, 0.9966)	0.9832/(0.9668, 0.9966)
	Swin Transformer	0.9763/(0.9593, 0.9932)	0.9768/(0.9609, 0.9904)	0.9763/(0.9593, 0.9932)	0.9764/(0.9595, 0.9932)
	ViT	0.9288/(0.8983, 0.9559)	0.9316/(0.9058, 0.9579)	0.9288/(0.8983, 0.9559)	0.9296/(0.8981, 0.9564)
	GoogLeNet	0.9831/(0.9661, 0.9966)	0.9835/(0.9685, 0.9966)	0.9831/(0.9661, 0.9966)	0.9831/(0.9666, 0.9966)
	MobileNetV2	0.9729/(0.9525, 0.9898)	0.9732/(0.9528, 0.9899)	0.9729/(0.9525, 0.9898)	0.9730/(0.9530, 0.9898)
Fold3	MaxViT	0.9831/(0.9661, 0.9966)	0.9835/(0.9678, 0.9966)	0.9831/(0.9661, 0.9966)	0.9831/(0.9665, 0.9966)
	ResNet18	0.9797/(0.9627, 0.9932)	0.9796/(0.9626, 0.9933)	0.9797/(0.9627, 0.9932)	0.9796/(0.9623, 0.9932)
	Swin Transformer	0.9492/(0.9254, 0.9729)	0.9490/(0.9218, 0.9728)	0.9492/(0.9220, 0.9729)	0.9491/(0.9222, 0.9728)
	ViT	0.9593/(0.9356, 0.9797)	0.9592/(0.9354, 0.9798)	0.9593/(0.9356, 0.9797)	0.9590/(0.9350, 0.9797)
	GoogLeNet	0.9797/(0.9627, 0.9932)	0.9804/(0.9647, 0.9934)	0.9797/(0.9627, 0.9932)	0.9798/(0.9629, 0.9932)
	MobileNetV2	0.9763/(0.9559, 0.9932)	0.9768/(0.9596, 0.9902)	0.9763/(0.9593, 0.9932)	0.9764/(0.9591, 0.9932)
Fold4	MaxViT	0.9898/(0.9762, 1.0000)	0.9898/(0.9765, 1.0000)	0.9898/(0.9762, 1.0000)	0.9898/(0.9763, 1.0000)
	ResNet18	0.9830/(0.9660, 0.9966)	0.9834/(0.9676, 0.9966)	0.9830/(0.9660, 0.9966)	0.9828/(0.9653, 0.9966)
	Swin Transformer	0.9864/(0.9728, 0.9966)	0.9866/(0.9728, 0.9966)	0.9864/(0.9728, 0.9966)	0.9864/(0.9727, 0.9966)
	ViT	0.9014/(0.8639, 0.9354)	0.9036/(0.8703, 0.9358)	0.9014/(0.8639, 0.9354)	0.9022/(0.8660, 0.9335)
	GoogLeNet	0.9694/(0.9490, 0.9864)	0.9723/(0.9563, 0.9870)	0.9694/(0.9490, 0.9864)	0.9698/(0.9500, 0.9898)
	MobileNetV2	0.9864/(0.9728, 0.9966)	0.9870/(0.9750, 0.9966)	0.9864/(0.9728, 0.9966)	0.9865/(0.9731, 0.9966)
Fold5	MaxViT	0.9728/(0.9524, 0.9898)	0.9736/(0.9556, 0.9899)	0.9728/(0.9524, 0.9898)	0.9730/(0.9530, 0.9898)
	ResNet18	0.9592/(0.9354, 0.9796)	0.9592/(0.9349, 0.9799)	0.9592/(0.9354, 0.9796)	0.9590/(0.9345, 0.9797)
	Swin Transformer	0.9558/(0.9320, 0.9762)	0.9560/(0.9320, 0.9767)	0.9558/(0.9320, 0.9762)	0.9559/(0.9315, 0.9764)
	ViT	0.9354/(0.9082, 0.9626)	0.9349/(0.9072, 0.9625)	0.9354/(0.9048, 0.9626)	0.9350/(0.9048, 0.9625)
	GoogLeNet	0.9660/(0.9422, 0.9864)	0.9660/(0.9451, 0.9864)	0.9660/(0.9422, 0.9864)	0.9660/(0.9451, 0.9863)
	MobileNetV2	0.9524/(0.9286, 0.9762)	0.9528/(0.9289, 0.9761)	0.9524/(0.9286, 0.9762)	0.9525/(0.9252, 0.9763)
Average	MaxViT	0.9817/(0.9647, 0.9952)	0.9821/(0.9663, 0.9953)	0.9817/(0.9647, 0.9952)	0.9818/(0.9650, 0.9952)
	ResNet18	0.9763/(0.9572, 0.9918)	0.9766/(0.9594, 0.9919)	0.9763/(0.9572, 0.9918)	0.9762/(0.9577, 0.9919)
	Swin Transformer	0.9688/(0.9498, 0.9864)	0.9689/(0.9493, 0.9853)	0.9688/(0.9484, 0.9864)	0.9688/(0.9483, 0.9864)
	ViT	0.9389/(0.9110, 0.9640)	0.9397/(0.9135, 0.9645)	0.9389/(0.9104, 0.9640)	0.9390/(0.9105, 0.9637)
	GoogLeNet	0.9763/(0.9572, 0.9918)	0.9771/(0.9603, 0.9920)	0.9763/(0.9572, 0.9918)	0.9764/(0.9582, 0.9925)
	MobileNetV2	0.9715/(0.9518, 0.9884)	0.9720/(0.9533, 0.9880)	0.9715/(0.9525, 0.9884)	0.9716/(0.9520, 0.9885)

When compared to the other analyzed designs, MaxViT’s better generalization capacity is evident from the testing results shown in Table 5. MaxViT consistently obtains the greatest accuracy values across folds, often above 0.98, with narrow 95% confidence intervals, indicating good statistical dependability and high discriminative power. This steady performance shows that MaxViT captures hierarchical and long-range relationships in knee MRI images better than traditional CNNs and other transformer-based models. MaxViT continues to be the most reliable and consistently top performing model, even though other convolution-based models like ResNet18, GoogLeNet, and MobileNetV2 also perform well. The ViT model, on the other hand, had much poorer accuracy and larger confidence ranges.

The confidence intervals shown in Table 5 offer crucial information about the robustness and statistical dependability of the assessed models. Low variability between testing folds is shown by narrow 95% confidence intervals, which imply that a model’s performance is steady and not excessively sensitive to the particular data split. MaxViT regularly shows among of the tightest confidence intervals in our analysis, indicating great stability and trustworthy generalization on unknown data. These narrow ranges suggest that MaxViT’s high accuracy scores are constant across various dataset subsets rather being an outcome of chance. The robustness of models like ResNet18, GoogLeNet, and Swin Transformer is further reinforced by their very narrow intervals. The ViT architecture, on the other hand, shows considerably larger confidence intervals, suggesting that its predictions are more uncertain and that performance fluctuated more between folds. This fluctuation implies that ViT could have difficulty identifying the pertinent structural patterns in the MRI data.

**Fig. 8:**
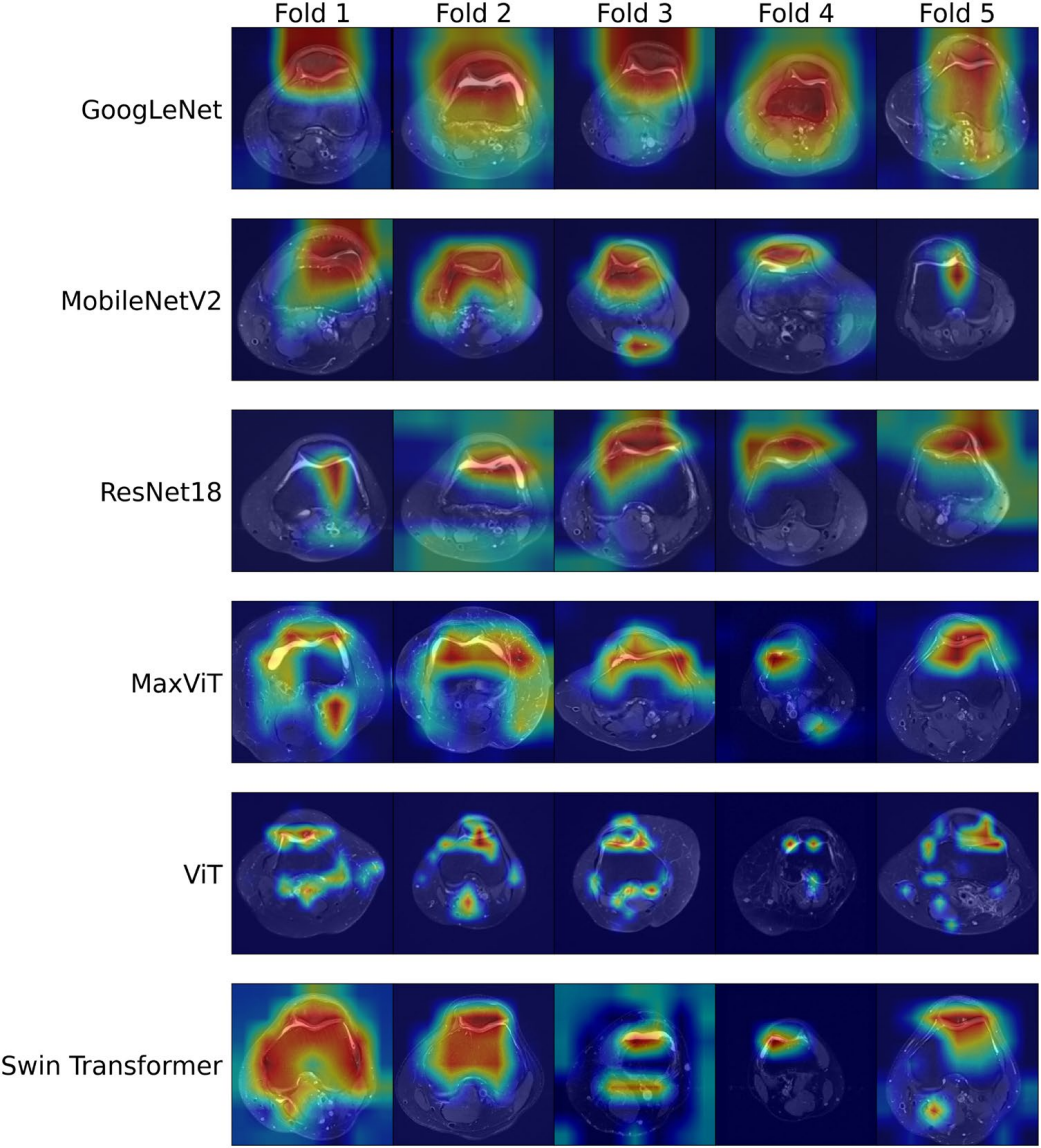
Grad-CAM visualizations of the models

Examples of Grad-CAM results for each model and fold are displayed in Fig. 8. All models consistently concentrate on the CMP and normal regions throughout each fold, according to an analysis of the Grad-CAM results.

The McNemar test p-values are presented in Table 6.

**Table 6:** McNemar test p-values for model comparisons against MaxViT

Fold	GoogLeNet	MobileNetV2	ResNet18	Swin Transformer	ViT
Fold1	1.000000	0.453125	1.000000	1.000000	0.581055
Fold2	1.000000	0.507812	1.000000	0.726562	0.000855
Fold3	1.000000	0.625000	1.000000	0.001953	0.039062
Fold4	0.109375	1.000000	0.687500	1.000000	0.000001
Fold5	0.625000	0.031250	0.218750	0.125000	0.003418

The McNemar test findings offer a statistical comparison of each model’s prediction behavior in relation to MaxViT when we examine Table 6. The p-values of GoogLeNet, MobileNetV2, ResNet18, and the Swin Transformer are all larger than 0.05 over the majority of folds, suggesting that there is no statistically significant difference between their misclassification patterns and MaxViT. In which means that these models do not exhibit statistically distinct error distributions in many folds, indicating that they frequently make mistakes on similar samples, even if MaxViT achieves better accuracy on average. However, the ViT consistently displays low p-values over several folds, all of which are below the 0.05 significance level. This suggests that the ViT’s misclassification behavior is very different from MaxViT’s.

Table 7 provides a performance comparison between the proposed model and the literature research.

**Table 7:** A comparison of the research gathered from the literature with the proposed model

Paper	Method	Objective	Modality	Accuracy
[5]	LSSVM	Knee disorders	VAG	0.9431
[19]	MLP	Osteoarthritis	VAG	0.893
[30]	CNN	Knee abnormalities	X-ray,CT,MRI	0.84
[40]	CNN	Osteoarthritis	VAG	0.936
[39]	Lasso Regression	CMP	MRI	0.70
Proposed	MaxViT	CMP	MRI	0.9817

[5] proposes an LSSVM model with an accuracy of 0.9431 for the classification of knee disorders using vibroarthrography signals, as Table 7 demonstrates. Similarly, [19] employs a multilayer perceptron model to detect osteoarthritis with an accuracy of 0.893 utilizing vibroarthrography data.

On the other hand, convolutional neural networks are employed in [30] and [40] to detect knee abnormalities. [30] integrates multi-modal data, including MRI, CT, and X-ray scans, to achieve an accuracy of 0.84. Conversely, [40] detects osteoarthritis from vibroarthrography signals with an accuracy of 0.936.

Furthermore, lasso regression is used by [39] to identify chondromalacia patella from MRI images, with a reported accuracy of 0.7. Comparatively, the suggested model, which uses MaxViT to classify chondromalacia patella from MRI data, performs better than current methods with an accuracy of 0.9817.

By identifying chondromalacia patella using MRI images, we advance the field as compared to previous research in the literature using our MaxViT-based approach. The outcomes show that our model outperforms earlier techniques in terms of accuracy, underscoring its potential to improve automated diagnosis of chondromalacia patella.

A thorough assessment of the literature revealed no research that uses MRI scans to classify chondromalacia patella. As a result, it was unable to compare directly with a research that used the same imaging modality and focused on the same illness. Results from clinically related studies that attempt to categorize pathological states of the knee joint using various modalities, such as vibroarthrography, are included in the literature comparison table given in this study. These studies may be useful to CMP in a wider clinical context. These comparisons are meant to draw attention to the absence of MRI-based methods and the gap in the literature surrounding CMP detection rather than to claim direct performance superiority. These studies are only considered contextual references due to the obvious distinctions in imaging method, illness focus, and clinical setting. However, the table has been kept to highlight the lack of automated MRI-based CMP classification methods and to show the present state of the field.

### Computation time

In this study, testing was conducted using the identical hardware specifications as were utilized for training.

Table 8 contains a list of all the parameters in the models.

**Table 8:** Number of the models’ parameters

Model	Parameters
GoogLeNet	5601954
MaxViT	30408650
MobileNetV2	2226434
ResNet18	11177538
Swin Transformer	27520892
ViT	85800194

The parameter counts of the assessed models are shown in Table 8, and the relationship between these parameters and computational complexity and predictive performance on the testing dataset is given in Table 9.

**Table 9:** Computation time comparison on testing dataset (seconds)

Fold	Batch Size	MaxViT	ResNet18	Swin Transformer	ViT	GoogLeNet	MobileNetV2
Fold1	1	12.16	3.14	6.77	7.71	9.15	3.74
	2	6.58	2.14	3.75	6.31	3.17	2.42
	4	4.38	1.80	3.17	5.70	2.04	1.72
	8	4.18	1.66	3.01	6.28	1.66	1.51
	16	4.09	1.58	2.93	5.00	1.53	1.41
	32	4.47	1.88	2.87	5.10	1.44	1.36
Fold2	1	13.23	3.08	7.27	7.16	5.09	3.75
	2	6.64	2.16	6.59	5.50	3.02	2.34
	4	4.34	1.84	4.52	4.92	2.02	1.70
	8	4.03	1.75	3.99	4.74	1.59	1.50
	16	3.95	1.69	3.88	5.97	1.63	1.41
	32	3.82	1.86	2.83	5.92	1.46	1.53
Fold3	1	12.48	5.96	6.84	7.95	6.23	3.95
	2	6.85	3.16	3.73	6.87	3.26	2.53
	4	4.46	1.91	3.04	5.24	1.88	1.89
	8	4.27	1.68	2.90	4.84	1.53	1.65
	16	4.02	1.65	2.91	4.82	1.37	1.57
	32	4.01	1.48	3.14	5.06	1.32	1.48
Fold4	1	12.48	3.03	6.75	7.77	9.98	3.74
	2	6.49	1.99	4.05	6.46	3.12	2.47
	4	4.28	1.64	3.13	5.20	2.03	1.81
	8	4.03	1.50	2.99	5.51	1.67	1.66
	16	4.04	1.45	2.93	5.59	1.55	2.58
	32	3.92	1.58	2.79	6.22	1.46	2.67
Fold5	1	12.44	3.02	6.69	9.64	7.69	4.03
	2	6.82	1.99	3.70	6.66	3.02	3.48
	4	4.39	1.66	3.03	6.14	2.17	2.46
	8	4.04	1.50	2.86	6.13	1.92	1.74
	16	3.94	1.44	2.79	5.59	1.54	1.59
	32	3.83	1.41	2.76	5.30	1.44	1.48
Average	1	12.56	3.65	6.86	8.05	7.63	3.84
	2	6.68	2.29	4.36	6.36	3.12	2.65
	4	4.37	1.77	3.38	5.44	2.03	1.92
	8	4.11	1.62	3.15	5.50	1.67	1.61
	16	4.01	1.56	3.09	5.39	1.52	1.71
	32	4.03	1.64	2.88	5.52	1.42	1.70

MobileNetV2 consistently shows the lowest computation time when the average values across all folds are analyzed, followed by GoogLeNet and ResNet18. Swin Transformer outperforms ViT among transformer-based models, however both models take longer to compute than traditional CNN designs. MaxViT exhibits much slower performance, especially at smaller batch sizes, and has the greatest testing time cost of all designs. All models experience the anticipated drop in calculation times as the batch size grows, however the pace of reduction varies according on the model design.

A thorough computational time study was carried out for both a batch size of one and many batch sizes in order to assess the model’s clinical relevance. An NVIDIA RTX 3050Ti with 4 GB of VRAM was utilized in this investigation; it was specifically selected as a low-cost substitute that is more likely to be found in clinical workstations than expensive, research grade hardware. As a result, the memory and computing time requirements were designed to represent a situation that is more similar to actual clinical settings. Batch sizes of 1, 2, 4, 8, 16, and 32 were used to measure and compare inference times for all models throughout the testing phase. This research made it possible to assess the models’ actual deployment elements, such as hardware compatibility and runtime efficiency, in addition to their theoretical performance. The findings show that all models may be implemented on inexpensive, common workstations that are probably used in clinical settings.

## Discussion

The study’s experimental results show that deep learning architectures, in particular the MaxViT, can perform better when it comes to automatically diagnosing chondromalacia patella from MRI images. The findings show that MaxViT outperforms CNN-based and transformer-based models, making it the best performing model across all assessed architectures. With an average testing accuracy of 0.9817, precision of 0.9821, recall of 0.9817, and F1-score of 0.9818, MaxViT demonstrated exceptional classification performance. These findings are a significant improvement above previous methods in the literature. MaxViT shows better classification performance than the lasso regression technique described by [39], which only obtained 0.7 accuracy for CMP diagnosis using MRI. This enhancement highlights the benefit of using MaxViT structures to capture intricate patterns in CMP images.

Additionally, MaxViT regularly showed 95% confidence intervals that were narrow. MaxViT’s statistical dependability suggests that it represents true generalization capabilities. No matter whether subset of data is utilized for testing, MaxViT retains strong performance, according to the confidence interval analysis. While the ViT consistently showed significantly different misclassification behavior over several folds with $p < 0.05$, the majority of CNN-based models did not reveal statistically significant variations in mistake patterns compared to MaxViT with $p > 0.05$. This implies that while pure transformer models struggle with various elements of the classification problem, MaxViT and CNN-based designs typically agree on challenging scenarios.

Examining Fig. 8, it is evident that all models concentrate on anatomically CMP related areas. This implies that rather than depending on dataset specific artifacts, the models capture significant disease related trends.

According to the parameter study, MaxViT achieves the best possible balance between computational efficiency and model capacity. MaxViT is significantly smaller than the ViT with about 30.4 million parameters, yet it performs noticeably better.

The suggested model is meant to serve as a decision-support tool rather than a stand-alone diagnostic system from a clinical standpoint. By functioning on regularly obtained knee MRI scans, it can be easily included into current radiological workflows without the need for extra imaging protocols or manual preprocessing. To automatically evaluate MRI studies after acquisition, the model might often be integrated into the picture archiving and communication system (PACS).

The suggested transformer-based MaxViT architecture demonstrates methodological features that are extremely relevant across a wide range of data limited and interpretation sensitive domains, in addition to its particular application to the diagnosis of chondromalacia patella.

Transformer models have shown superior performance in myocardial ischemia detection from electrocardiography signals, as thoroughly reviewed by [2]. This highlights the usefulness of attention mechanisms for modeling long range dependencies in time series physiological data. This evidence implies that the attention strategies used in MaxViT are suitable for sequential signal processing as well as static imaging.

The MicroCrystalNet architecture, which was suggested for micro-porosity mapping in geological formations, shows how well thought out convolutional and attention inspired mechanisms can achieve strong performance and high interpretability in the geoscience domain even with limited data availability [45]. Similar to this, [44] demonstrated how cross-modal feature learning techniques may successfully handle sparse data conditions in the classification of paleontological fossils, highlighting the significance of multi-scale and attention-based representations in situations when labeled examples are hard to come by.

Dual-stage feature fusion techniques have been applied in sustainable energy monitoring to combine red-green-blue (RGB) and temperature data for photovoltaic defect identification under practical limitations [9]. Similar functions are provided by MaxViT’s hierarchical block and grid attention structure, which allows for selective emphasis on diagnostically significant regions while suppressing unnecessary information. The significance of parameter efficient designs for complex physical systems has also been highlighted in computational fluid dynamics, where lightweight architectures like FluidNet-Lite have shown that effective neural representations can successfully model multiphase flow in porous media without unnecessary computational overhead [43].

When combined, these cross-domain investigations show that rather than being a task specific solution, the suggested MaxViT-based framework represents a generalizable methodological paradigm. Its interpretability, scalability, and capacity to manage small amounts of training data through effective attention mechanisms point to possible applications to a variety of application domains. These include more general applications in infrastructure analysis and environmental monitoring, as well as structural engineering tasks like wireless coverage prediction from architectural layouts [6].

## Conclusion

The MaxViT is a very successful design for the automated identification of chondromalacia patella from MRI images, as this study shows. MaxViT continuously obtained state-of-the-art classification performance by thorough examination throughout 5-fold cross validation. These outcomes far outperform both transformer models and conventional CNN-based designs.

With narrow confidence intervals and positive McNemar test results, MaxViT demonstrated exceptional stability and statistical reliability in addition to great predictive accuracy. MaxViT’s inference performance is still well within acceptable bounds for real-time clinical decision assistance, although requiring a little more processing time than lightweight CNNs.

The use of a comparatively limited dataset gathered from a single institution is one of the study’s primary drawbacks. The model’s high accuracy may have been influenced by scanner settings, quality of image, and imaging practices unique to the institution. As a result, rather than learning actual disease-related characteristics, the model might have acquired dataset or institution-specific patterns. Because external data from other institutions may cause the good performance reported in this study to decline, it should be viewed cautiously.

Furthermore, the near perfect accuracy findings might look statistically overoptimistic due to the testing set’s small size and lack of variety. Therefore, inter-institutional validation studies encompassing various MRI scanners, patient groups, and acquisition techniques are essential to accurately evaluate the model’s generalizability under actual clinical situations.

Building bigger, multi center datasets, creating models for four grade CMP classification, and methodically testing the model on separate external datasets will be the main goals of future research. It is anticipated that these actions will improve generalizability and offer a more trustworthy evaluation of the model’s actual clinical deployment potential.

## Data Availability

The data used in this study are not publicly available due to privacy restrictions and institutional policies.
